# In vitro study of the replication capacity of the RGNNV and the SJNNV betanodavirus genotypes and their natural reassortants in response to temperature

**DOI:** 10.1186/1297-9716-45-56

**Published:** 2014-05-20

**Authors:** Valentina Panzarin, Elisabetta Cappellozza, Marzia Mancin, Adelaide Milani, Anna Toffan, Calogero Terregino, Giovanni Cattoli

**Affiliations:** 1 Istituto Zooprofilattico Sperimentale delle Venezie, OIE Reference Laboratory for Viral Encephalopathy and Retinopathy, Viale dell’Università 10, 35020 Legnaro, PD, Italy

## Abstract

Betanodaviruses are the causative agents of viral nervous necrosis and affect a broad range of fish species worldwide. Their bi-segmented genome is composed of the RNA1 and the RNA2 molecules encoding the viral polymerase and the coat protein, respectively. In southern Europe the presence of the RGNNV and the SJNNV genotypes, and the RGNNV/SJNNV and RGNNV/SJNNV reassortants has been documented. Several studies have reported a correlation between water temperature and disease onset. To explore the replication efficiency of betanodaviruses with different genomes in relation to temperature and to understand the role of genetic reassortment on viral phenotype, RGNNV, SJNNV, RGNNV/SJNNV and RGNNV/SJNNV field isolates were fully sequenced, and growth curves generated in vitro at four different temperatures (15, 20, 25, 30 °C) were developed for each isolate. The data obtained, corroborated by statistical analysis, demonstrated that viral titres of diverse betanodavirus genotypes varied significantly in relation to the incubation temperature of the culture. In particular, at 30 °C betanodaviruses under investigation presented different phenotypes, and viruses containing the RNA1 of the RGNNV genotype showed the best replication efficiency. Laboratory results demonstrated that viruses clustering within the same genotype based on the polymerase gene, possess similar growth kinetics in response to temperature, thus highlighting the key role of RNA1 in controlling viral replication at different environmental conditions. The results generated might have practical implications for the inference of viral phenotype according to genetic features and may contribute to a better understanding of betanodavirus ecology.

## Introduction

The increasing amount of genetic information obtained from viral genomes sequencing, aids research into the exploration of the genotype-phenotype relationships to determine the genetic traits responsible for different phenotypic features, which in turn may have practical implications for disease recognition and control. Nevertheless, the phenotype of a virus is not only dependent on its “intrinsic” genetic features (e.g. specific mutations, reassortment, recombination) but it is also governed by “extrinsic” variables [1-4]. Among these, temperature is certainly one of the most important environmental factors in determining the ecological and physiological status of viruses hosted by poikilothermic animals such as fish, whose thermoregulation systems are generally absent or extremely rudimentary. Fish body temperature varies with that of their environment, and consequently aquatic viruses need to adapt to a wide range of temperatures to be able to replicate at different conditions.

Viruses within the genus *Betanodavirus* are the causative agents of a highly infectious fish disease known as viral nervous necrosis (VNN), also known as viral encephalopathy and retinopathy (VER). The genome of betanodaviruses is composed of two single stranded positive sense RNA molecules. The RNA1 segment encodes the RNA-dependant RNA polymerase (RdRp), or protein A, and gives rise to the RNA3 sub-genomic transcript which is translated into proteins B1 and B2, whilst the RNA2 segment encodes the coat protein (CP) [5-8]. The phylogenetic analysis of the RNA2 genetic segment allowed the identification of four different genotypes: striped jack nervous necrosis virus (SJNNV), tiger puffer nervous necrosis virus (TPNNV), barfin flounder nervous necrosis virus (BFNNV), red-spotted grouper nervous necrosis virus (RGNNV) [9]. A putative fifth genotype isolated from *Scophthalmus maximus* named as turbot nervous necrosis virus (TNNV) is awaiting for official classification [10]. Iwamoto et al. [11] demonstrated that the degree of viral replication in SSN-1 monolayers and the severity of the cytopathic effect (CPE) may vary according to the genotype and to the incubation temperature of the cultures. It has been also observed that optimal culture temperatures vary among genotypes: 15–20 °C for the BFNNV genotype, 20 °C for the TPNNV genotype, 20–25 °C for the SJNNV genotype and 25–30 °C for the RGNNV genotype [12]. More recent studies have highlighted that it is the RGNNV genotype that can replicate in vitro at the widest range of temperatures, from a minimum of 15 °C to a maximum of 35 °C [13,14]. Noteworthy, betanodaviruses are widely distributed worldwide in cold, temperate and tropical climate zones. Generally speaking, temperature dependency of betanodaviruses seems to correspond to their geographic distribution. To date, the TPNNV genotype has been described only once in Japan [9]. Cold water betanodaviruses grouping within the BFNNV genotype have been reported in Norway, France, the UK, eastern Canada and in the north-east of the USA [15-21]. The SJNNV genotype distribution appears confined to Spain and Japan [9,22-25]. Conversely, as a result of viral adaptation to different temperatures, the RGNNV seems to be the most widely diffused genotype, extending to Asia, Africa, Australia and several other Mediterranean areas and, accordingly, it is able to infect a large variety of warm water finfish species [23,24,26-33]. Together with the RGNNV and the SJNNV genotypes, the circulation of reassortant viruses in the form of the RGNNV/SJNNV, harbouring the RNA1 of the RGNNV and the RNA2 of the SJNNV, and the SJNNV/RGNNV, harbouring the SJNNV-RNA1 and the RGNNV-RNA2, have also been reported in southern Europe [23,29,32]. To date, the biological and ecological properties of these viruses have been poorly described, and little is known about the role of genetic reassortment and its effects on viral phenotype.

In order to identify the genetic regions involved in temperature dependency of piscine nodaviruses, the infectious RNA transcription system established by Iwamoto et al. [34] has recently been applied to produce artificial RGNNV and SJNNV viruses and their reassortants. The study has demonstrated that both genetic segments are involved in determining temperature sensitivity of betanodaviruses; however, the RNA1 molecule is capable of regulating this process independently from RNA2, confirming the observation that viral replication is vulnerable to temperature variations [35]. Although the reverse genetics technology provides a suitable experimental model for studying reassortant betanodaviruses, no information is available for natural field strains. For the first time, we have investigated the role of genetic reassortment in naïve betanodaviruses, as well as the genotype-phenotype relation as a function of temperature. For this purpose, natural reassortant RGNNV/SJNNV and SJNNV/RGNNV strains and RGNNV and SJNNV genotypes were genetically characterized and cultivated in cell monolayers at different incubation temperatures to assess their replication efficiency. Experimental data were validated through extensive statistical analysis.

## Materials and methods

### Virus strains and propagation in cell culture

On the basis of previous phylogenetic analysis of partial RNA1 and RNA2 sequences [23,36], four betanodavirus isolates representative of the RGNNV (283.2009), SJNNV (484.2.2009), RGNNV/SJNNV (367.2.2005) and SJNNV/RGNNV (389/I96) genetic variants were selected for further genetic and phenotypic characterization (Table 1). All but one of the selected viruses (283.2009; 367.2.2005; 389/I96) were originally isolated from the same fish species (sea bass, *Dicentrarchus labrax*). However, strain 484.2.2009 isolated from a Senegalese sole (*Solea senegalensis*) was also included in the selection, because to date no SJNNV infection has been reported in sea bass.

**Table 1:** Betanodavirus isolates used in this study for genetic and phenotypic characterization

**Isolate**	**Genotype**	**Host**	**Origin**	**Reference**	**GenBank accession no.**	
					**RNA1**	**RNA2**
283.2009	RGNNV	*Dicentrarchus labrax*	Italy	[23]	JN189865	JN189992
484.2.2009	SJNNV	*Solea senegalensis*	Spain	[23]	JN189814	JN189919
367.2.2005	RGNNV/SJNNV	*Dicentrarchus labrax*	Italy	[23]	JN189909	JN189936
389/I96	SJNNV/RGNNV	*Dicentrarchus labrax*	Italy	[46]	KF386163	KF386164

Betanodavirus isolates were propagated in E-11 cell monolayers (ECACC no. 01110916; [12]) incubated at 25 °C in Leibovitz medium (L-15) (Sigma-Aldrich, St. Louis, MO, USA), supplemented with 10% FCS, L-Glutamine (2 mM) and antibiotics (100 IU/mL penicillin, 100 μg/mL streptomycin and 0.25 μg/mL amphotericin B) [37]. Inoculated cell cultures were checked daily for cytopathic effect (CPE). Supernatants were collected upon disruption of cell monolayers, clarified by centrifugation at 4000 × *g* for 15 min at 4 °C, and subsequently subjected to further investigations.

### RNA isolation and identification of the 3’ and 5’ terminal sequences

For each virus, total RNA was extracted from 100 μL of cell culture supernatant using the NucleoSpin® RNA II (Macherey–Nagel GmbH & Co, Düren, Germany), and subsequently treated with Tobacco Acid Pyrophosphatase (TAP) (Epicentre Biotechnologies, Madison, WI, USA) for 5’-cap removal. The 3’ and 5’ ends were ligated with the T4 RNA Ligase 1 (New England BioLabs, Ipswich, MA, USA) in a 25 μL reaction mix. Briefly, 15 μL of de-capped RNA were incubated for 5 min at 65 °C with 20U rRNasin RNase Inhibitor (Promega, Fitchburg, WI, USA), 1X T4 RNA Ligase Reaction Buffer, 10% dimethyl sulfoxide (DMSO) and water, and subsequently cooled on ice for 2 min. The reaction mix was then incubated for 60 min at 37 °C with 20U of rRNasin RNase Inhibitor (Promega) and 20U of T4 RNA Ligase 1 (New England BioLabs). Enzymes were inactivated by incubating the reaction mix for 10 min at 65 °C. Circular RNA was subjected to reverse transcription (RT) for cDNA synthesis by using the SuperScript® III Reverse Transcriptase (Life Technologies, Grand Island, NY, USA) according to the manufacturer’s instructions and adding 10% DMSO to the reaction mix. PCR was subsequently carried out in a final volume of 25 μL containing 2 μL of template cDNA, 1X Cloned Pfu DNA polymerase reaction buffer, 240 μM dNTPs, 1.25U Pfu Turbo DNA polymerase (Agilent Technologies, Santa Clara, CA, USA) and 0.2 μM specific forward and reverse primer hybridizing respectively upstream the 3’ end and downstream the 5’ end. Primer sequences are available upon request. The cDNA was denaturated at 95 °C for 2 min, and 40 cycles of the following conditions were applied: denaturation at 95 °C for 45 s, annealing at 50 °C for 1 min, elongation at 72 °C for 2 min. The amplification was completed with 2 min elongation at 72 °C. PCR products were subjected to electrophoresis in agarose gel, and were subsequently purified using the QIAquick Gel Extraction Kit (Qiagen, Hilden, Germany). The sequences of the 3’-5’ junctions were generated using the BigDye Terminator v3.1 cycle sequencing kit (Life Technologies). The products of sequencing reactions were cleaned up using the Performa DTR Ultra 96-Well kit (Edge BioSystems, Gaithersburg, MD, USA) and sequenced in a 16-capillary ABI PRISM 3130×l Genetic Analyzer (Life Technologies). Sequencing data were assembled and edited with the SeqScape® software v2.5 (Life Technologies).

### Complete genome sequencing and phylogenetic characterization

In order to obtain complete genetic data related to the four selected betanodavirus strains, the nucleotide sequences corresponding to the full length RNA1 and RNA2 were determined for each virus. Previously de-capped RNA was subjected to RT by using the High Capacity cDNA Reverse Transcription Kit (Life Technologies) following the manufacturer’s instructions. The identification of the 3’ and 5’ terminal sequences allowed to design of specific primer sets for the whole genome amplification. PCR and sequencing were carried out as described above. Primer sequences can be provided on request.

The obtained sequences were aligned and compared to reference betanodavirus sequences publicly available in GenBank using the MEGA 4 package [38]. For both the RNA1 and RNA2 genetic segments, maximum likelihood (ML) phylogenetic trees were estimated using the best-fit general time-reversible (GTR) model of nucleotide substitution with gamma-distributed rate variation among sites, and a heuristic SPR branch swapping search available in PhyML version 3.0 [39]. Bootstrap resampling (100 replications) assessed the robustness of individual nodes of the phylogeny. Pairwise nucleotide identities estimated among the RNA1 and RNA2 of the four betanodavirus strains herein described and the two reference strains SGWak97 [GenBank: AY324869; AY324870] and SJNag93 [GenBank: AB056571; AB056572] were determined.

### Experimental design

E-11 cells were grown in 5 mL culture flasks with Leibovitz medium (L-15) (Sigma-Aldrich), supplemented with 10% FCS, L-Glutamine (2 mM) and antibiotics (100 IU/mL penicillin, 100 μg/mL streptomycin and 0,25 μg/mL Amphotericin B). Cell monolayers were infected in three replicates with each of the isolates at a multiplicity of infection (MOI) of 1.0. After 1 h adsorption at 25 °C, all the *inocula* were brought to the same volume with L-15 medium without growth factor (FCS). Inoculated monolayers were then incubated at 15, 20, 25 and 30 °C and checked regularly for CPE. A volume of 700 μL was sampled from each flask at 0 (T_0_), 20 (T_1_), 30 (T_2_), 45 (T_3_), 55 (T_4_), 69 (T_5_), 79 (T_6_), 93 (T_7_), 117 (T_8_), 141 (T_9_) and 165 (T_10_) hours post infection (hpi), and subsequently replaced with an equal amount of medium. Collected cell culture supernatants were subjected to viral titration in E-11 monolayers by endpoint dilutions assays. Viral titres were calculated according to the Spearman-Karber formula [40]. Finally, average titres expressed as TCID_50_/mL were calculated among replicates, and were used for developing growth curves for each incubation temperature.

### Statistical analysis

The linear mixed model (LMM) for longitudinal data [41] was used to investigate the influence of genotype, temperature, exposure time and their possible interactions on viral replication efficiency. Different types of models with fixed and random effects were analysed according to the experimental design. To assess the repeatability of the experiment, replicates were tested as fixed effect, in addition to genotype, exposure time, temperature and their interactions. In this model it was assumed that each replicate was independent. Secondly, a linear mixed model was tested, where genotype, exposure time, temperature and their interaction were considered as fixed effects, while replicate was considered as a random effect. This model assumed constant variance among replicates of the same virus and among observations within the same replicate. The latter model was further developed considering different structures of variance and covariance matrix (VAR/COV) to take into account the existence of possible correlations (ρ) among repeated measurements of the same replicate. The structures of VAR/COV were: compound symmetry (CS), heterogeneous compound symmetry (CSH), unstructured (UN), first order autoregressive (AR(1)). *P*-values < 0.10 were considered significant. Graphical Student residuals analysis (residuals versus predicted plot, residuals distribution and residuals Q-Q plot) and tests for residuals normality (Shapiro Wilks and Kolmogorov Smirnov tests) were used to verify the goodness of the proposed models [41]. SAS software 9.3 was used to carry out the statistical analyses (PROC MIXED function) [42,43].

## Results

### Genetic characterization and phylogenetic analysis

**Figure 1:**
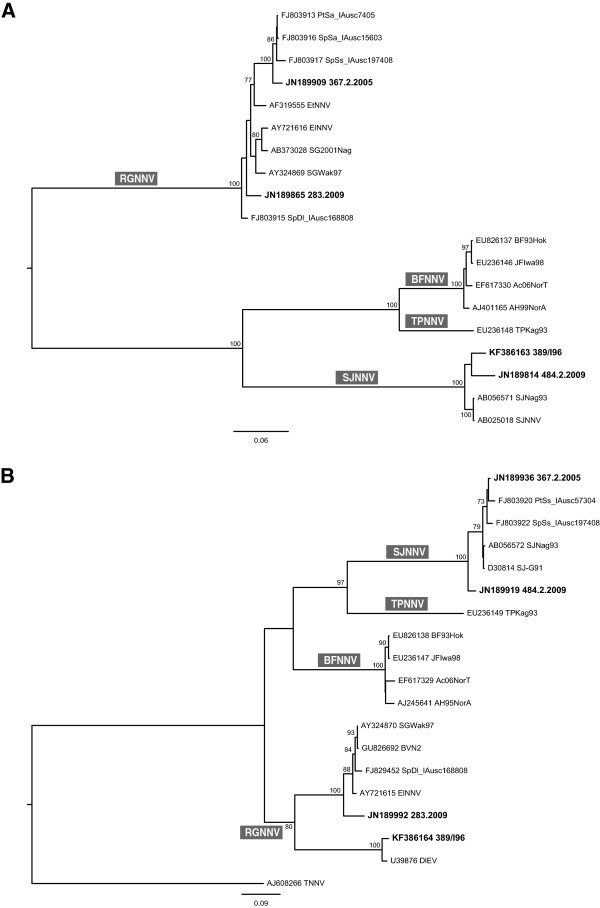
**ML phylogenetic trees. (A)** RNA1 complete ORF. **(B)** RNA2 complete ORF. Fish nodaviruses characterized in the present study are labelled in bold. The numbers at branch points correspond to bootstrap values expressed as percentages. The genotype subdivision according to Nishizawa et al. [9] is shown at the main branches. Scale bars represent nucleotide substitutions per site.

The RNA1 genetic segment differed in length among the four betanodavirus strains. The 5’-untranslated regions (UTRs) were 78 nt long, whereas the 3’-UTRs varied from 75 to 77 nt. Strains 283.2009 and 367.2.2005 showed a 2949 nt ORF, corresponding to 983 aa, while the RNA1 coding region of samples 484.2.2009 and 389/I96 was 3 nt longer, determining an amino acid insertion in the RdRp at position 888. The RNA2 5’-UTRs and 3’-UTRs varied from 26 to 27 nt and from 370 to 390 nt, respectively. The complete ORF for strains 283.2009 and 389/I96 was 1017 nt, encoding a coat protein of 339 amino acids. Viruses 484.2.2009 and 367.2.2005 presented an insertion of 6 nt within the T4 variable region [44], corresponding to 341 amino acids capsid protein. The deduced lengths of RNA3 5’-UTRs and 3’-UTRs for the four betanodavirus strains spanned from 23 to 26 nt and from 122 to 124 nt respectively. RNA3 complete ORFs was 228 nt long, encoding 76 aa B2 protein.The phylogenetic analysis based on the complete ORFs of RNA1 and RNA2 genetic segments confirmed that the 283.2009 and the 484.2.2009 isolates are indeed RGNNV-type and SJNNV-type respectively, and corroborated the identification of the RGNNV/SJNNV and SJNNV/RGNNV reassortants (samples 367.2.2005 and 389/I96, respectively) (Figure 1).

Nucleotide and amino acid similarities calculated for the RNA1 and the viral polymerase are 96.4% and 98% between strains 283.2009 and 367.2.2005, and 97.2% and 98.3% between strains 484.2.2009 and 389/I96. The RNA1 nucleotide sequence of the RGNNV and the RGNNV/SJNNV viruses was 97.6 and 96.7% identical to that of strain SGWak97 (corresponding to 99.3 and 97.6% amino acid identity). The polymerase gene of the SJNNV and of the SJNNV/RGNNV viruses was 97.1 and 97.8% identical to that of strain SJNag93 (corresponding to 97.8 and 98.1% amino acid identity). Pairwise similarity was calculated also for the RNA1 nucleotide region spanning from position 84 to position 1419 and putatively responsible for betanodavirus temperature sensitivity [35], which corresponds to the polymerase N-terminal. Nucleotide identity was 96.1% between strains 283.2009 and 367.2.2005 (corresponding to 98.4% amino acid identity) and 97.7% between strains 484.2.2009 and 389/I96 (corresponding to 97.7% amino acid identity). When considering the 84–1419 nucleotide region, the SGWak97 strain was 97.3 and 96.7% identical to samples 283.2009 and 367.2.2005 (100 and 98.4% amino acid identity, respectively), and the SJNag93 strain was 97.2 and 97.8% identical to samples 484.2.2009 and 389/I96 (96.8 and 97.9% amino acid identity, respectively). Finally, nucleotide and amino acid identities calculated for the RNA2 and the coat protein were 88.1% and 89.2% between strains 283.2009 and 389/I96, and 97.3% and 97% between strains 484.2.2009 and 367.2.2005. The SGWak97 strain was 97 and 88.1% identical to samples 283.2009 and 389/I96 (96.3 and 87.9% amino acid identity, respectively), and the SJNag93 strain was 97.8 and 99.1% identical to samples 484.2.2009 and 367.2.2005 (97 and 98.8% amino acid identity, respectively).

### Phenotypic characterization

Inoculated cells were inspected regularly for the appearance of CPE (Table 2). At 15 °C, E-11 monolayers were characterized by cell shrinkage, most likely due to the suboptimal incubation temperature for their cultivation. From 93 hpi (T_7_) onwards, an alteration of all the infected monolayers was observable, where cells appeared dark and contracted and tended to detach from the surface of the flask. However, no specific lesions related to betanodavirus infection (i.e. vacuolization of cells) were detected and none of the four viruses determined the complete disruption of cell monolayers at this temperature. At 20 °C, no CPE was noticed at the early stages of the infection. However, at 69 hpi (T_5_) all the betanodavirus strains under investigation showed typical cellular vacuoles which evolved into extended *foci* of rounded, granular and vacuolated cells after 93 hpi (T_7_). All the viral strains determined the complete disruption of cell monolayers by 117 hpi (T_8_). At 25 °C, the RGNNV-type, the SJNNV-type and the two reassortants showed the same phenotype: after 69 hpi (T_5_) the presence of diffuse CPE was observed, resulting in the disruption of the monolayers at 93 hpi (T_7_). Interestingly, clear phenotypic differences among the four betanodavirus strains were noticed at 30 °C. In particular, the RGNNV strain and the RGNNV/SJNNV reassortant determined an early appearance of CPE just after 45 hpi (T_3_), which became more severe at 69 hpi (T_5_). The complete disruption of cell monolayer was observable after 93 hpi (T_7_). Noteworthy, the SJNNV strain and the SJNNV/RGNNV reassortant showed a completely different phenotype. In particular, the SJNNV/RGNNV strain determined the appearance of diffuse vacuoles after 69 hpi (T_5_), while no CPE was observable for the SJNNV isolate. By 93 hpi (T_7_) both the SJNNV strain and the SJNNV/RGNNV reassortant induced the emergence of multiple *foci* of vacuolated cells. CPE severity worsened over time, but none of these strains determined the complete disruption of the cell monolayers by the end of the experiment.

**Table 2:** Cytopathic effect (CPE) observed in E-11 cell monolayers infected with RGNNV, SJNNV, RGNNV/SJNNV, SJNNV/RGNNV betanodavirus strains at different incubation temperatures

	**15 °C**				**20 °C**				**25 °C**				**30 °C**			
	**RGNNV**	**SJNNV**	**RGNNV/SJNNV**	**SJNNV/RGNNV**	**RGNNV**	**SJNNV**	**RGNNV/SJNNV**	**SJNNV/RGNNV**	**RGNNV**	**SJNNV**	**RGNNV/SJNNV**	**SJNNV/RGNNV**	**RGNNV**	**SJNNV**	**RGNNV/SJNNV**	**SJNNV/RGNNV**
**T1**	**§**	**§**	**§**	**§**	**-**	**-**	**-**	**-**	**-**	**-**	**-**	**-**	**-**	**-**	**-**	**-**
**T3**	**§**	**§**	**§**	**§**	**-**	**-**	**-**	**-**	**-**	**-**	**-**	**-**	**+ + +**	**-**	**+ + +**	**-**
**T5**	**§**	**§**	**§**	**§**	**+**	**+**	**+**	**+**	**+ +**	**+ +**	**+ +**	**+ +**	**+ + + +**	**-**	**+ + + +**	**+**
**T7**	*	*	*	*	**+ + + +**	**+ + + +**	**+ + + +**	**+ + + +**	**+ + + + +**	**+ + + + +**	**+ + + + +**	**+ + + + +**	**+ + + + +**	**+ +**	**+ + + + +**	**+ +**
**T8**	*	*	*	*	**+ + + + +**	**+ + + + +**	**+ + + + +**	**+ + + + +**						**+ + +**		**+ + +**
**T9**	*	*	*	*										**+ + + +**		**+ + + +**
**T10**	*	*	*	*										**+ + + +**		**+ + + +**

Data were recorded at T_1_ (20 hpi), T_3_ (45 hpi), T_5_ (69 hpi), T_7_ (93 hpi), T_8_ (117 hpi), T_9_ (141 hpi) and T_10_ (165 hpi). Cells appearance, estimation of the CPE and its degree of severity were assessed according to the following levels: cell shrinkage (§); alteration of the cell monolayer characterized by dark and contracted cells with the tendency to detach from the surface of the flask (*); absence of CPE (-); cellular alteration and presence of diffuse vacuoles (+); presence of diffuse *foci* of rounded, granular and vacuolated cells (+ +); presence of diffuse *foci* of rounded, granular and vacuolated cells and initial cell monolayer disruption (+ + +); presence of diffuse *foci* of rounded, granular and vacuolated cells and advanced cell monolayer disruption (+ + + +); complete cell monolayer disruption (+ + + + +).

### Growth kinetics

**Figure 2:**
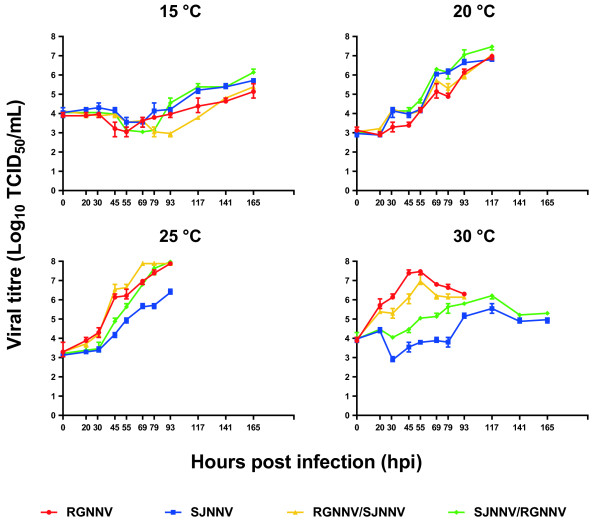
**Betanodavirus replication kinetics.** Growth curves developed for the RGNNV, SJNNV, RGNNV/SJNNV and SJNNV/RGNNV strains at different incubations temperatures (15, 20, 25, 30 °C). Numbers in the *y*-axis represent the Log_10_ of viral titres expressed as TCID_50_/mL; in the *x*-axis, the observation points expressed as hours post infection (hpi) are reported. Data are plotted as mean and range of triplicate samples.

The average titres determined in culture supernatant samples collected at different time points were used to develop graphs describing the growth kinetics of the RGNNV and the SJNNV genotypes and their natural reassortants at different incubation temperatures (Figure 2). At 15 °C, a similar growth was noted for all the viruses, with a slight and slow increase of viral titre over time, although strains SJNNV and SJNNV/RGNNV seemed to have a better fitness, particularly towards the end of the experiment. In general, the chronic trend of viral growth indicates that this temperature is suboptimal for betanodavirus replication. The higher titres were recorded after 165 hpi (RGNNV: 10^5.1^ TCID_50_/mL; SJNNV: 10^5.7^ TCID_50_/mL; RGNNV/SJNNV: 10^5.3^ TCID_50_/mL; SJNNV/RGNNV: 10^6.1^ TCID_50_/mL). At 20 °C, all the viruses displayed comparable kinetics. However, this temperature showed a higher compatibility with an improved replication fitness, determining a regular increase of viral titre over time, particularly for strains SJNNV and SJNNV/RGNNV. The higher titres were obtained at 117 hpi (RGNNV: 10^6.9^ TCID_50_/mL; SJNNV: 10^6.8^ TCID_50_/mL; RGNNV/SJNNV: 10^7.0^ TCID_50_/mL; SJNNV/RGNNV: 10^7.4^ TCID_50_/mL). At 25 °C, the RGNNV strain and the two reassortants showed an acute growth trend characterized by a rapid and efficient replication, yielding high titres (10^7.9^ TCID_50_/mL) after 93 hpi. However, this condition was suboptimal for the SJNNV strain, which showed a slow growth and had a lower titre (10^6.3^ TCID_50_/mL after 93 hpi). Interestingly, at 30 °C the differences in replication efficiency among strains became striking. RGNNV and RGNNV/SJNNV rapidly multiplied during the first hours of incubation, reaching the peak of replication (10^7.5^ TCID_50_/mL and 10^7.3^ TCID_50_/mL, respectively) only after 55 hpi. The acute phase was followed by a progressive decrease in viral titres, until the cell monolayer was completely disrupted. On the contrary, the SJNNV and the SJNNV/RGNNV betanodaviruses showed a chronic growth trend, characterized by a slow and poor replication. The higher titres were obtained at 117 hpi (SJNNV: 10^5.5^ TCID_50_/mL; SJNNV/RGNNV: 10^6.2^ TCID_50_/mL).

### Statistical analysis

Given the diverse phenotypes and growth kinetics of viral strains at different incubation temperatures, data related to the observation points T_9_ (141 hpi) and T_10_ (165 hpi) at 20 °C and T_8_ (117 hpi), T_9_ (141 hpi) and T_10_ (165 hpi) at 25 °C are missing for all viruses, as well as data associated with T_8_ (117 hpi), T_9_ (141 hpi) and T_10_ (165 hpi) at 30 °C for viruses RGNNV and RGNNV/SJNNV. This is why statistical analysis was performed using a reduced dataset, so as to have the same number of exposure times (up to T_7_, corresponding to 93 hpi) for each temperature and genotype.

The first tested model, where replicates were considered as fixed effects, demonstrated the repeatability of the experiment, highlighting that differences in titres measurements among different replicates are not significant.

The best LMM selected for longitudinal data presented genotype, exposure time, temperature and their interaction as fixed effects while the replicate was considered as a random effect, with a AR(1) structure of VAR/COV matrix (ρ = 0.18). Graphical Student residuals analysis, Shapiro Wilks and Kolmogorov Smirnov tests of posterior analysis, confirmed the goodness of the proposed model (Additional file 1).

The analysis indicated that variables genotype, exposure time, temperature and their interactions were significant to explain the trend of the titres and highlighted the existence of differences among the increase of viral titres over time, depending on the temperature. In detail, the analysis showed that at 15 °C viral titres of strains RGNNV, SJNNV and SJNNV/RGNNV increased significantly more than RGNNV/SJNNV titres over time. Furthermore, no difference existed between the replication efficiency of pairs RGNNV and SJNNV, RGNNV and SJNNV/RGNNV, SJNNV and SJNNV/RGNNV over time. At 20 °C, no significant difference was observed between the increase of viral titres of strains RGNNV and RGNNV/SJNNV, and strains SJNNV and SJNNV/RGNNV over time, while isolates SJNNV and SJNNV/RGNNV showed a significantly higher replication efficiency when compared to strain RGNNV and strain RGNNV/SJNNV. At 25 °C and 30 °C, SJNNV viral titres increased significantly less than RGNNV, RGNNV/SJNNV and SJNNV/RGNNV titres over time. However, at both temperatures no significant differences were noticeable among the replication efficiencies of viruses RGNNV, RGNNV/SJNNV and SJNNV/RGNNV.

In order to evaluate whether the statistical observations might have been biased by the use of a restricted dataset, additional analyses were performed considering all the observation points up to 165 hpi for strains RGNNV, SJNNV, RGNNV/SJNNV and SJNNV/RGNNV at 15 °C, and for viruses SJNNV and SJNNV/RGNNV at 30 °C. The model developed for 15 °C showed no significant differences in the replication fitness of strains RGNNV, SJNNV, RGNNV/SJNNV and SJNNV/RGNNV. At 30 °C, the difference in terms of replication efficiency observed between strains SJNNV and SJNNV/RGNNV in the time slot T_0_-T_7_ was not significant when considering the entire duration of the experiment (up to T_10_ corresponding to 165 hpi).

Generally speaking, strains RGNNV and RGNNV/SJNNV showed similar growth trends at 15, 20, 25 and 30 °C. Similarly, the SJNNV and the SJNNV/RGNNV viruses exhibited comparable replication fitness at all the temperatures considered. Differences in viral titres between pairs RGNNV-RGNNV/SJNNV and SJNNV-SJNNV/RGNNV became more evident at 30 °C.

## Discussion

Betanodavirus natural infections can occur at different water temperatures, depending on the genotype. The association between the onset of VNN and environmental conditions has been documented in several papers [45,46]. A number of experimental trials have also demonstrated the effect of temperature, infectious dose and viral multiplication rate on betanodavirus pathogenicity and disease course [36,47-49]. These observations have led to the assumption that betanodavirus replication is most likely a temperature-sensitive process, as previously hypothesized also by Hata et al. [35]. With the aim of shedding light into the complex interplay existing between betanodavirus genetic features, environmental conditions and viral replication capacity, the present study investigates the effect of temperature on the in vitro replication of naïve RGNNV and SJNNV strains and on natural reassortants. We observed that all the viruses barely grew at 15 °C, while the rise of the incubation temperature up to 20 and 25 °C resulted in a boost of their multiplication capacity. The only exception was strain SJNNV, which showed a reduced fitness at 25 °C. A sharp increase in viral titre of the RGNNV and the RGNNV/SJNNV strains was noticeable at 30 °C, while the SJNNV and the SJNNV/RGNNV viruses showed a suboptimal growth kinetics comparable to that observed at 15 °C. Remarkably, viruses possessing the polymerase gene of the same genotype exhibited comparable replication trends, and strains with the RNA1 segment of the RGNNV genotype efficiently multiplied at higher temperatures (30 °C). All these data confirm that the RNA1 genetic segment and its encoded protein play a major role in controlling temperature sensitivity of fish nodaviruses, substantiating previous findings by Hata et al. [35]. Nevertheless, a possible role of the RNA2 and the RNA3 cannot be ruled out a priori although, to the best of the authors’ knowledge, there is no evidence for the involvement of these molecules in the regulation of betanodavirus replication.

Interesting observations resulted from the sequencing analysis carried out in this study. Within the amino acid region 1–445 which controls betanodavirus temperature dependency [35], four transmembrane domains (TMDs) with moderate level of hydrophobicity were previously identified for the Greasy Grouper Nervous Necrosis Virus (GGNNV, belonging to the RGNNV genotype) [50] and the Atlantic Halibut Nodavirus (AHNV, grouping within the BFNNV genotype) [51]. These TMDs are located at positions 1–40, 225–246 for the AHNV, and at positions 153–173, 229–249 for the GGNNV. All but the TMD 153–173 were confirmed to contain mitochondrial targeting signals, responsible for the localization of protein A within the mitochondria membrane and for the formation of the replication complex. Interestingly, three putative TMDs located at positions 6–26, 152–173 and 224–249 were identified for the RGNNV, SJNNV, RGNNV/SJNNV and the SJNNV/RGNNV viruses herein characterized. TMD 224–249 was not predicted for strain SJNNV (data not shown), which notably replicates less efficiently at 25 and 30 °C. Furthermore, when comparing the RGNNV, SJNNV, RGNNV/SJNNV, SJNNV/RGNNV, SGWak97 and SJNag93 sequences corresponding to the TMDs identified by Guo et al. and Mézeth et al. [50,51], 9 amino acid signatures characteristic for each genotype were identified at positions 7, 19, 155, 223, 232, 235, 241, 251 and 254. In detail, 4 out of 9 signatures determined a dramatic change in the physical-chemical properties of the amino acids: Ala_7-RGNNV_ vs Glu_7-SJNNV_; Met_223-RGNNV_/Leu_223-RGNNV_ vs Lys_223-SJNNV_; Thr_241-RGNNV_ vs Leu_241-SJNNV_; Pro_251-RGNNV_ vs Gln_251-SJNNV_. Positive-strand RNA viruses commonly associate their polymerase and viral RNA to the membranes of cellular organelles to replicate their genomes [52]. Several members of the *nodaviridae* family, namely AHNV, Flock house virus (FHV), GGNNV, Nodamura virus, Wuhan Nodavirus (WhNV), have shown to replicate in the mitochondria [50,51,53-55]. It is reasonable to speculate that the four betanodavirus strains herein described possess the same replication strategy, while the existence of specific amino acid signatures might relate with differences in growth kinetics of betanodaviruses with diverse genomes. The role of these mutations in determining protein A localization, membrane affinity, viral RNA recruitment, stability and accumulation as well as protein A interaction with host cell proteins is an issue which certainly deserves further investigations.

Results on viral replication obtained in this study were fully corroborated by extensive statistical analysis, and substantiate previous findings reported by Hata et al. [35], achieved by using the reverse genetics technique. Importantly, despite the methodological limits of our study due to the use of naïve reassortant strains, which have shown a certain degree of genetic diversity if compared to the parental RGNNV and SJNNV, the study of natural viruses give the most truthful picture of betanodavirus phenotype in response to temperature and definitively clarify the effect of genetic reassortment on viral replication. Interestingly, in our previous work we discovered that the polymerase gene evolves more rapidly than the viral capsid gene [23]. Whether this is a consequence of betanodavirus adaptive mechanisms to different climate and environmental conditions mainly regulated by RNA1, it is still an open question.

Recently, Vendramin et al. [36] have compared the pathogenicity of ten different betanodavirus strains, including samples 283.2009 (RGNNV), 484.2.2009 (SJNNV), 367.2.2005 (RGNNV/SJNNV) and 389/I96 (SJNNV/RGNNV). Sea bass juveniles were bath challenged at 20 °C, and were subsequently subjected to gradually increasing water temperatures (23 and 25 °C). Overall, it was observed that the mortality rate proportionally increased with water temperature. Noteworthy, strain 283.2009 (RGNNV) was the most lethal virus, reassortant strains 389/I96 and 367.2.2005 increased their pathogenicity at 23 and 25 °C respectively, and virus 484.2.2009 (SJNNV) was lethal for fish at 20 °C without any further increase in pathogenicity at higher temperatures. Generally speaking, the in vivo results obtained by Vendramin et al. are in agreement with the outcomes of the present study, and the mortality rate determined by different betanodavirus strains seems to reflect their in vitro multiplication capacity at 20 and 25 °C, suggesting that replication efficiency is crucial for betanodavirus pathogenicity. However, our results indicate that although both the SJNNV/RGNNV and the SJNNV strains induced CPE at 30 °C but not at 15 °C, each virus reached nearly identical titres when the two incubation temperatures were compared. This means that for the SJNNV/RGNNV and the SJNNV genotypes the appearance of CPE is not exclusively dose-dependent, which suggests that temperature might influence viral phenotype through different mechanisms other than replication efficiency.

Data gained in the present study might have practical implications, as they could help infer the viral phenotype on the basis of the genetic data. Furthermore, the in vitro characterization of the viral phenotype appears to be a suitable methodology for the prediction of betanodavirus pathogenicity under controlled environmental conditions, and might have important applications in fish farming and vaccinology. Indeed, Nishizawa et al. [49] applied the principle of regulating viral pathogenicity by controlling fish rearing temperature, and immunized sevenband grouper (*Epinephelus septemfasciatus*) with a live vaccine strain by keeping the water temperature at 17 °C. This vaccination strategy, despite the obvious limitations for application in open farms or sea cages, might be used inside hatcheries or, alternatively, during transportation of fish to reduce the volume of vaccine needed [56]. Additionally, the identification of viruses which replicate more efficiently in vitro may assist in selecting a candidate vaccine strain suitable for high-throughput antigen production.

## Abbreviations

VNN: viral nervous necrosis; VER: viral encephalopathy and retinopathy; RdRp: RNA-dependant RNA polymerase; CP: coat protein; SJNNV: Striped jack nervous necrosis virus; TPNNV: Tiger puffer nervous necrosis virus; BFNNV: Barfin flounder nervous necrosis virus; RGNNV: Red-spotted grouper nervous necrosis virus; TNNV: Turbot nervous necrosis virus; SSN-1: Striped snakehead cell line; FCS: Foetal calf serum; CPE: Cytopathic effect; TAP: Tobacco acid pyrophosphatase; DMSO: Dimethyl sulfoxide; RNA: Ribonucleic acid; RT: Reverse transcription; cDNA: Complementary deoxyribonucleic acid; PCR: Polymerase chain reaction; dNTPs: Deoxinucleotides; ML: Maximum likelihood; GTR: General time-reversible; MOI: Multiplicity of infection; TCID: Tissue culture infectious dose; LMM: Linear mixed model; VAR/COV: Variance and covariance matrix; CS: Compound symmetry; CSH: Heterogeneous compound symmetry; UN: Unstructured, AR(1): first order autoregressive; AR(1): First order autoregressive; UTRs: Untranslated regions; ORF: Open reading frame; TMD: Transmembrane domain; GGNV: Greasy grouper nervous necrosis virus; AHNV: Atlantic halibut nodavirus; FHV: Flock house virus; WhNV: Wuhan nodavirus.

## Competing interests

The authors declare that they have no competing interests.

## Authors’ contributions

GC conceived the study and coordinated the work described. VP, AT, CT were involved in the experimental design. EC, VP, AM performed the experiments and interpreted the results. MM carried out the statistical analysis. VP wrote the manuscript. GC, AT, CT were involved in the interpretation of the results and critically read the manuscript. All authors read and approved the final manuscript.

## Supplementary Material

Additional file 1 **Graph Student residual analysis: residuals versus predicted plot, residuals distribution and residuals Q-Q plot.** Residuals without a particular trend, with normal distribution and good alignment over line indicate that the model is correct. The hypothesis of normal distribution of residuals is further tested using the Shapiro-Wilk and Kolmogorov-Smirnov test. Value of *p* > 0.10 indicates that the residual has a normal distribution. Click here for file
