# Supplementary figures and images for: In vitro study of the replication capacity of the RGNNV and the SJNNV betanodavirus genotypes and their natural reassortants in response to temperature
Source: Vet Res. 2014 May 20;45(1):56. doi: 10.1186/1297-9716-45-56 (PMC4050099; doi:10.1186/1297-9716-45-56)

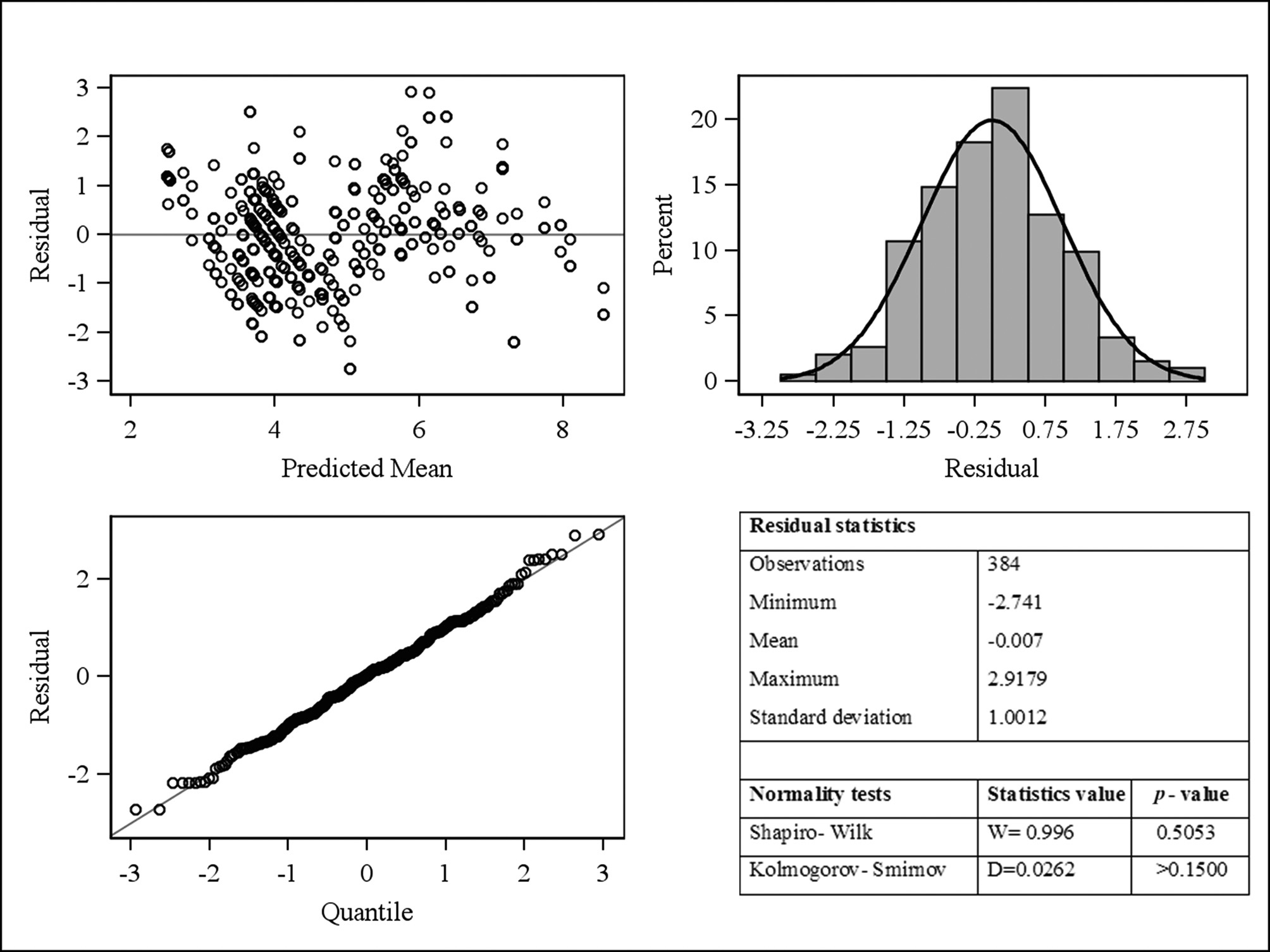

Supplement: Additional file 1 — Graph Student residual analysis: residuals versus predicted plot, residuals distribution and residuals Q-Q plot. Residuals without a particular trend, with normal distribution and good alignment over line indicate that the model is correct. The hypothesis of normal distribution of residuals is further tested using the Shapiro-Wilk and Kolmogorov-Smirnov test. Value of p > 0.10 indicates that the residual has a normal distribution. [file 1297-9716-45-56-S1.jpeg]
